# Research Progress on Main Symptoms of Novel Coronavirus Pneumonia Improved by Traditional Chinese Medicine

**DOI:** 10.3389/fphar.2020.556885

**Published:** 2020-09-11

**Authors:** Chuan-hong Luo, Le-le Ma, Hui-min Liu, Wei Liao, Run-chun Xu, Zhi-min Ci, Jun-zhi Lin, Li Han, Ding-kun Zhang

**Affiliations:** ^1^ School of Pharmacy, State Key Laboratory of Characteristic Chinese Drug Resources in Southwest China, Chengdu University of Traditional Chinese Medicine, Chengdu, China; ^2^ Central Laboratory, Teaching Hospital of Chengdu University of Traditional Chinese Medicine, Chengdu, China

**Keywords:** COVID-19, traditional Chinese medicine, fever, cough, fatigue, diarrhea

## Abstract

Novel coronavirus (COVID-19) pneumonia has become a major threat to worldwide public health, having rapidly spread to more than 180 countries and infecting over 1.6 billion people. Fever, cough, and fatigue are the most common initial symptoms of COVID-19, while some patients experience diarrhea rather than fever in the early stage. Many herbal medicine and Chinese patent medicine can significantly improve these symptoms, cure the patients experiencing a mild 22form of the illness, reduce the rate of transition from mild to severe disease, and reduce mortality. Therefore, this paper summarizes the physiopathological mechanisms of fever, cough, fatigue and diarrhea, and introduces Chinese herbal medicines (Ephedrae Herba, Gypsum Fibrosum, Glycyrrhizae Radix et Rhizoma, Asteris Radix et Rhizoma, Ginseng Radix et Rhizoma, Codonopsis Radix, Atractylodis Rhizoma, *etc.*) and Chinese patent medicines (Shuang-huang-lian, Ma-xing-gan-shi-tang, *etc.*) with their corresponding therapeutic effects. Emphasis was placed on their material basis, mechanism of action, and clinical research. Most of these medicines possess the pharmacological activities of anti-inflammatory, antioxidant, antiviral, and immunity-enhancement, and may be promising medicines for the treatment or adjuvant treatment of COVID-19 patients.

## Introduction

Novel coronavirus (Corona Virus disease 2019, COVID-19) pneumonia has become a massive threat to global public health. It is highly infectious, with a relatively high mortality rate, causing a sharp increase in the number of infections in a short period. Of even greater concern, is that some people infected with COVID-19 do not have obvious symptoms in the initial stage, and become potential super-communicators. More than 80,000 COVID-19 cases have been confirmed in China so far. Surprisingly, by the end of April, 2020, novel infections were almost zero, the country was recovering, and the epidemic in China was nearing completion. However, the virus is still causing panic around the world. The Director-General of the World Health Organization (WHO) declared that COVID-19 can be characterized as a pandemic on March 11, 2020 (World Health Organization, 2020). The epidemic is spreading rapidly in Italy, the United States, Spain, Germany, Iran, France, South Korea, Japan, and other countries. More than 1.6 billion confirmed cases and 650,000 cumulative deaths have been reported worldwide as of July 28, 2020. Although most of these countries have a well-developed medical and health service system, they are caught in the dilemma of shortage and exhaustion of public medical resources. At the same time, they are facing a sharp increase in the number of patients, which will lead to a series of serious consequences, such as a severe shortage of timely medical treatment, a high incidence of transition from mild to severe disease, an increasing mortality of the severely-affected patients, and a large-scale infection of medical staff. Therefore, it is of great value and significance to share the treatment experience of China in anti-epidemic to all countries in the world, so that more infected people can get treatment, and a greater public health crisis can be avoided.

At present, the transmission characteristics and clinical symptoms of COVID-19 have been relatively fully recognized. It is infectious and can be transmitted through respiratory droplets, digestive tracts, and contact; and the population is generally susceptible (Guan and Xian, 2020; Medical Administration Bureau, 2020). The incubation period is about 7 days on average and up to 14 days. Fever, dry cough, and fatigue are the main clinical manifestations. Half of the patients developed dyspnea after 8 days. Severe patients rapidly progressed to acute respiratory distress syndrome (ARDS), septic shock, metabolic acidosis, and coagulation dysfunction that are difficult to correct (Huang et al., 2020). However, there is still a lack of effective means of treatment. The research and development cycle of new drugs and vaccines is too long, so it is the first choice to seek effective treatment strategies in the existing treatment methods.

The COVID-19 belongs to the scope of “Wen Yi” in traditional Chinese medicine (TCM). And TCM has unique cognition and rich experience in diagnosis and treatment of “Wen Yi”. The integration of traditional Chinese and western medical treatments played a unique role in the prevention and treatment of SARS in 2003 (Zhang et al., 2004). It may be one of the reasons that the mortality rate in Mainland China (7%, 349/5327) was lower than that in Hong Kong, (17%, 299/1755), Taiwan (11%, 37/346), or even in the world (9.6%, 774/8096) (Wu et al., 2008). Dr. J. Kenneth Baillie, a member of the WHO panel on clinical management for COVID-19, suggested that corticosteroid treatment should be avoided, and argued that steroids have little benefit to patients, with harm outweighing the benefit. He proposed that clinicians may give priority to symptomatic and supportive treatment (Russell et al., 2020), which is highly consistent with the concept of syndrome differentiation and treatment in TCM. According to the clinical observation of 34 cases carried out by Professor Zhang Boli and others in the Wuhan Jiangxia makeshift hospital, the disappearance rate of other concomitant symptoms, the clinical cure rate, and the incidence of common type patient to severe type in the integrated group were respectively 85.3%, 91.2%, and 5.9%. Compared with conventional western medical therapy, treatment with TCM was significantly better than those in the western medicine group (38.9%, 61.1%, and 33.3%). It was found that the treatment of COVID-19 using an integration of TCM and western medicine may significantly relieve the clinical symptoms, shorten the course of the disease, and improve the clinical cure rate, which is superior to the results using western medicine alone (Xia et al., 2020). Moreover, the participation of TCM in all provinces of China is as high as 90%, which has demonstrated that TCM has made an important contribution to the prevention and control of this epidemic.

**Figure 1:**
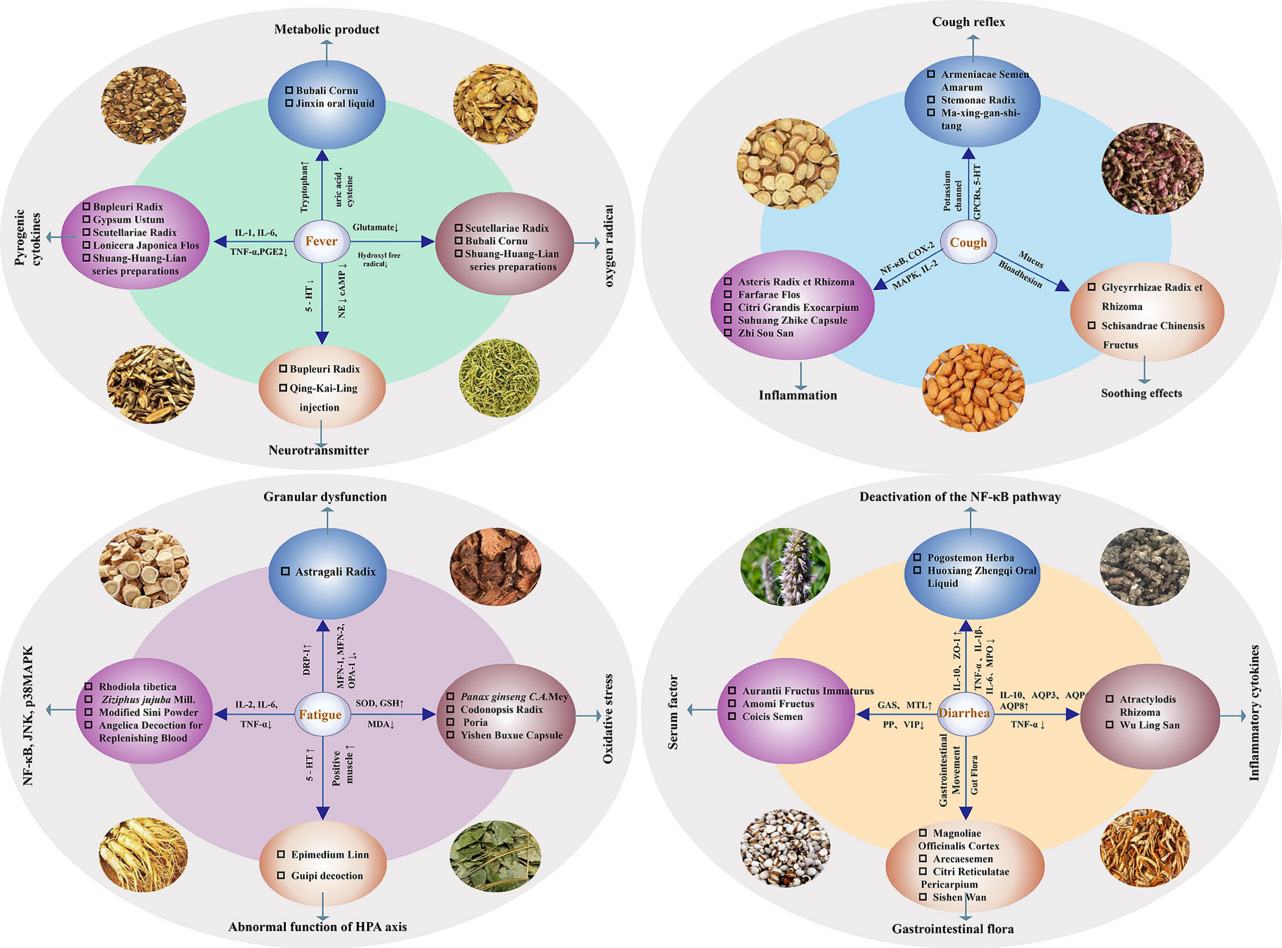
Therapeutic effect of Chinese Herbal Medicine on the main symptoms (fever, cough, fatigue and diarrhea) of COVID-19 in the early stage.

Therefore, this review aimed to summarize various Chinese herbal medicines (**Table 1**, **Figure 1**) and Chinese patent medicines that have properties which would be beneficial in treating symptoms associated with coronavirus infection (fever, cough, fatigue and diarrhea). Additionally, evidence quality evaluation criteria were established to select references suitable for this study, as shown in **Table 2**. Based on the existing literature, we sought drugs with scientific evidence that improve the clinical manifestations of patients with COVID-19, which may provide supplementary and alternative treatments to underdeveloped or medically under-resourced areas. More importantly, we hope to explore the potential drugs for COVID-19 and provide novel ways and ideas for the prevention and treatment of COVID-19.

**Table 1:** Descriptive table of the Chinese herbal medicines mentioned in this paper.

Number	Scientific name	Latin name	Common name	Local Chinese name	Parts used
1	*Acorus tatarinowii* Schott	Acori Tatarinowii Rhizoma	Grassleaf sweetflag rhizome	Shi-chang-pu	Rhizome
2	*Alisma orientale* (Sam.) Juz.	Alismatis Rhizoma	—	Ze-xie	Rhizome
3	*Amomum villosum* Lour.*, Amomum villosum* Lour. var. *xanthioides* (Wall. ex Baker) T.L.Wu & S.J.Chen, *Amomum longiligulare* T.L.Wu	Amomi Fructus	Villous amomum fruit	Sha-ren	Fruit
4	*Angelica dahurica* (Hoffm.) Benth. & Hook.f. ex Franch. & Sav., *Angelica dahurica* var. *formosana* (Boissieu) Yen	Angelicae Dahuricae Radix	Dahurian angelica root	Bai-zhi	Root
5	*Angelica sinensis* (Oliv.) Diels	Angelicae Sinensis Radix	Chinese angelica root	Dang-gui	Root
6	*Arctium lappa* L.	Arctii Fructus	Great burdock achene	Niu-bang-zi	Fruit
7	*Areca catechu* L.	Arecaesemen	Areca seed	Bing-lang	Seed
8	*Areca catechu* L.	Arecae Pericarpium	Areca peel	Da-fu-pi	Pericarpium
9	*Aster tataricus* L. f.	Asteris Radix	Aster root	Zi-wan	Root
10	*Atractylodes lancea* (Thunb.) DC., *Atractylodes chinensis* (DC.) Koidz.	Atractylodis Rhizoma	Atractylodes	Cang-zhu	Rhizome
11	*Atractylodes macrocephala* Koidz.	Atractylodis Macrocephalae Rhizoma	Largehead atractylodes rhizome	Bai-zhu	Rhizome
12	*Aucklandia lappa* DC.	Aucklandiae Radix	Common aucklandia root	Mu-xiang	Root
13	*Bubalus bubalis* Linnaeus	Bubali Cornu	Buffalo horn	Shui-niu-jiao	Horn
14	*Bupleurum chinense* DC. and *Bupleurum scorzonerifolium* Willd.	Bupleuri Radix	Chinese thorowax root	Chai-hu	Root
15	*Chaenomeles speciosa* (Sweet) Nakai	Chaenomelis Fructus	Common floweringqince fruit	Mu-gua	Fruit
16	*Cinnamomum cassia* (L.) J.Presl	Cinnamomi Ramulus	Cassia twig	Gui-zhi	Branch
17	*Cinnamomum cassia* (L.) J.Presl	Cinnamomi Cortex	Cassia bark	Rou-gui	Bark
18	*Citrus × aurantium* L., *Citrus sinensis* (L.) Osbeck	Aurantii Fructus Immaturus	—	Zhi-shi	Fruit
19	*Citrus grandis* ‘Tomentosa’, *Citrus grandis* (L.) Osbeck	Citri Grandis Exocarpium	Pummelo peel	Hua-ju-hong	Pericarpium
20	*Citrus medica* L.	Citri Sarcodactylis Fructus	Finger citron	Fo-shou	Fruit
21	*Citrus reticulata* Blanco	Citri Reticulatae Pericarpium	Dried tangerine peel pericarpium	Chen-pi	Pericarpium
22	*Codonopsis pilosula* (Franch.) Nannf., *Codonopsis pilosula* Nannf.var.modesta (Nannf.) L.T.Shen, *Codonopsis tangshen* Oliv.	Codonopsis Radix	Tangshen	Dang-shen	Root
23	*Coix lacryma-jobi* var. *ma-yuen* (Rom.Caill.) Stapf	Coicis Semen	—	Yi-yi-ren	Seed
24	*Cryptotympana pustulata* Fabricius	Cicadae Periostracum	Cicada slough	Chan-tui	Slough
25	*Cuscuta australis* R.Br., *Cuscuta chinensis* Lam.	Cuscutae Semen	Dodder seed	Tu-si-zi	Seed
26	*Cynanchum stauntonii* (Decne.) Schltr. ex H.Lév., *Cynanchum glaucescens* (Decne.) Hand.-Mazz.	Cynanchi Stauntonii Rhizoma et Radix	Willowleaf	Bai-qian	Root and rhizome
27	*Descurainia Sophia* (L.) Webb. ex Prantl	Descurain Semen	Pepperweed seed	Ting-li-zi	Seed
28	*Dimocarpus longan* Lour.	Longan Arillus	Langan aril	Long-yan-rou	Aril
29	*Dryopteris crassirhizoma* Nakai	Dryopteridis Crassirhizomatis Rhizoma	Male fern rhizome	Mian-ma-guan-zhong	Rhizome
30	*Ephedra sinica* Stapf, *Ephedra intermedia* Schrenk et C. A. Mey and *Ephedra equisetina* Bunge	Ephedrae Herba	Ephedra aerial parts	Ma-huang	Aerial parts
31	*Epimedium brevicomu* Maxim., *Epimedium sagittatum* (Siebold & Zucc.) Maxim., *Epimedium pubescens* Maxim., *Epimedium koreanum* Nakai	Epimedii Folium	—	Yin-yang-huo	Leaf
32	*Eriobotrya japonica* (Thunb.) Lindl.	Eriobotryae Folium	Loquat leaf	Pi-pa-ye	Leaf
33	*Eucommia ulmoides* Oliv.	Eucommiae Cortex	Eucommia bark	Du-zhong	Bark
34	*Euodia rutaecarpa* (Juss.) Benth., *Euodia rutaecarpa* (Juss.) Benth. var. *officinalis* (Dode) Huang, *Euodia rutaecarpa* (Juss.) Benth. var. *bodinieri* (Dode) Huang	Euodiae Fructus	Medicinal evodia fruit	Wuzhuyu	Fruit
35	*Fritillaria cirrhosa* D.Don, *Fritillaria unibracteata* P.K.Hsiao & K.C.Hsia, *Fritillaria przewalskii* Maxim. ex Batalin., *Fritillaria delavayi* Franch., *Fritillaria taipaiensis* P.Y.Li, *Fritillaria unibracteata* var. wabuensis (S.Y.Tang & S.C.Yueh) Z.D.Liu, Shu Wang & S.C.Chen	Fritillariae Cirrhosae Bulbus	Fritillaria bulb	Chuan-bei-mu	Bulb
36	*Fritillaria usuriensis* Maxim.	Fritillariae Ussuriensis Bulbus	—	Ping-bei-mu	*Bulbus*
37	*Gardenia jasminoides* J.Ellis	Gardeniae Fructus	—	Zhi-zi	Fruit
38	*Glycine max* (L.) Merr.	Sojae Semen Praeparatum	Fermented soybean	Dan-dou-chi	Seed
39	*Glycyrrhiza uralensis* Fisch., *Glycyrrhiza inflata* Batalin*, Glycyrrhiza glabra* L.	Glycyrrhizae Radix et Rhizoma	Licorice root	Gan-cao	Root and rhizome
40	Gypsum Fibrosum	Gypsum Fibrosum	Gypsum	Shi-gao	
41	*Houttuynia cordata* Thunb.	Houttuyniae Herba	—	Yu-xing-cao	Herb
42	*Hyriopsis cumingii* (Lea), *Cristaria plicata* (Leach), *Pteria martensii* (Dunker)	Margaritifera Concha	Margaritifera	Zhen-zhu-mu	Shell
43	*Isatis indigotica* Fortune ex Lindl.	Isatidis Radix	Isatis root	Ban-lan-gen	Root
44	*Ligusticum chuanxiong* Hort.	Chuanxiong Rhizoma	Szechwan lovage rhizome	Chuan-xiong	Rhizome
45	*Lonicera japonica* Thunb.	Lonicera Japonica Flos	Honeysuckle flower	Jin-yin-hua	Flower
46	*Lophatherum gracile* Brongn.	Lophatheri Herba	Lophatherum herb	Dan-zhu-ye	Stem and leaf
47	*Lycium barbarum* L.	Lycii Fructus	Arbary wolfberry fruit	Gou-qi-zi	Fruit
48	*Magnolia officinalis* Rehder & E.H.Wilson, *Magnolia officinalis* var. *biloba* Rehder & E.H.Wilson	Magnoliae Officinalis Cortex	Officinal magnolia bark	Hou-po	Bark
49	*Mentha haplocalyx* Briq.	Menthae Haplocalycis Herba	Peppermint	Bo-he	Aerial parts
50	Mongolian *Astragalus membranaceus* (Fisch.) Bge.var.mongholicus (Bge.) Hsiao, *Apodium Astragalus membranaceus* (Fisch.) Bge.	Astragali Radix	Milkvetch root	Huang-qi	Root
51	*Morus alba* L.	Mori Cortex	White mulberry root-bark	Sang-bai-pi	Root-bark
52	*Myristica fragrans* Houtt.	Myristicae Semen	Nutmeg seed	Rou-dou-kou	Seed
53	*Paeonia lactiflora* Pall.	Paeoniae Radix Alba	White peony root	Bai-shao	Root
54	*Panax ginseng* C.A.Mey.	Ginseng Radix Et Rhizoma	Ginseng root	Ren-shen	Root and rhizome
55	*Perilla frutescens* (L.) Britton	Perillae Folium	—	Zi-su	Leaf
56	*Perilla frutescens* (L.) Britton	Perillae Fructus	—	Zi-su-zi	Fruit
57	*Peucedanum praeruptorum* Dunn	Peucedani Radix	—	Qian-hu	Root
58	*Pheretima aspergillum* (E.Perrier)*, Pheretima vu1garis* Chen, *Pheretima guillelmi* (Michaelsen), *Pheretima pectinifera* Michaelsen	Pheretima	Earthworm	Di-long	Body
59	*Phragmites communis* Trin.	Phragmitis Rhizoma	Reed rhizome	Lu-gen	Rhizome
60	*Pinellia ternate* (Thunb.) Makino	Pinelliae Rhizoma	Pinellia tuber	Ban-xia	Tuber
61	*Platycodon grandiflorus* (Jacq.) A. DC.	Platycodonis Radix	Platycodon root	Jie-geng	Root
62	*Pogostemon Cablin* (Blanco) Benth.	Pogostemonis Herba	Cablin patchouli herb	Guang-huo-xiang	Aerial parts
63	*Polygala tenuifolia* Willd.	Polygalae Radix	—	Yuan-zhi	Root
64	*Polygonum cuspidatum* Siebold & Zucc.	Polygoni Cuspidati, Rhizoma Et Radix	Giant knotweed rhizome	Hu-zhang	Root and rhizome
65	*Polyporus umbellatus* (Pers.) Fries	Polyporus	—	Zhu-ling	Sclerotium
66	*Poria cocos* (Schw.) Wolf	Poria	Indian bread	Fu-ling	Sclerotium
67	*Prunus armeniaca* L.*, Prunus sibirica* L*., Prunus mandshurica* (Maxim.) Koehne	Armeniacae Semen Amarum	Apricot kernel	Ku-xing-ren	Seed
68	*Psoralea corylifolia* L.	Psoraleae Fructus	Malaytea scurfpea fruit	Bu-gu-zhi	Fruit
69	*Rehmannia glutinosa* (Gaertn.) DC.	Rehmanniae Radix	Rehmannia root	Di-huang	Root
70	*Rheum palmatum* L., *Rheum tanguticum* Maxim.ex B*a*lf.*, Rheum officinale* Baill.	Rhei Radix Et Rhizoma	Rhubarb	Da-huang	Root and rhizome
71	*Rhodiola crenulata* (Hook. f. & Thoms.) H. Ohba	Rhodiolae Crenulatae Radix et Rhizoma	Rhodiolae root	Hong-jing-tian	Root and rhizome
72	*Schisandra Chinensis* (Turcz.) Baill.	Schisandrae Chinensis Fructus	Schisandra fruit	Wu-wei-zi	Fruit
73	*Schizonepeta tenuifolia* (Benth) Briq.	Schizonepetae Herba	Fineleaf schizonepeta herb	Jing-jie	Aerial parts
74	*Scutellaria baicalensis* Georgi	ScutellariaeRadix	Baical skullcap root	Huang-qin	Root
75	*Stemona sessilifolia* (Miq.) Miq., *Stemona japonica* (Blume) Miq, *Stemona tuberosa* Lour.	Stemonae Radix	Stemona root	Bai-bu	Root
76	*Tussilago farfara* L	Farfarae Flos	Coltsfoot flower	Kuan-dong-hua	Flower
77	*Ziziphus jujuba* Mill.	Ziziphus Jujuba	Chinese date	Da-zao	Fruit
78	*Ziziphus jujube* Mill. var. *spinosa* (Bunge) HuexH.F.Chou	Ziziphi Spinosae Semen	Spine date seed	Suan-Zao-ren	Seed

**Table 2:** Evidence quality evaluation criteria.

Type	Evidence degree	Treatment
Clinical trials	Ia	Meta-analyses of randomized controlled trials
	Ib	Evidence from randomized controlled trial (n ≥ 50)
	Ic	Evidence from randomized controlled trial (n ≥ 20)
	IIa	Evidence from well-performed nonexperimental descriptive studies as well as comparative studies, correlation review studies, network pharmacology studies and case- studies
Animal trials	I	Evidence from *in vivo* experiments with reasonable groups (multi-dose, positive and negative control group, n ≥ 8) and credible results
	II	Evidence from *in vivo* experiments with reasonable groups (a single dose, positive and negative control group, n ≥ 5) and credible results
	III	Evidence from *in vivo* experiments with relatively reasonable groups (a single dose, n ≥ 5) and credible results
	IV	Evidence from *in vitro* experiments with credible results

Evidence below this criterion will be excluded.

## Fever

### The Mechanism of Fever

Fever is known as a characteristic defensive host mechanism, consisting of an increase in body temperature, occurring in response to various types of infectious or non-infectious stimuli(Aryal et al., 2019). Based on guidelines for the management of febrile illnesses provided by authorities such as the WHO and the Society of Critical Care Medicine and the Infectious Disease Society of America, among others, equivalent rectal temperature of ≥ 38°C or axillary temperatures of ≥ 37.5°C are indicative of fever in both adults and children (Ogoina, 2011). Fever is not only a disease, but also an important clinical manifestation of many diseases (Dewitt et al., 2017). One of the important clinical manifestations of COVID-19 is fever. From January 1 to January 28, 2020, 136 (98.6%) of 138 consecutive confirmed COVID-19 patients in the Central South Hospital of Wuhan University in China had clinical manifestations of fever. Early fever generally lasts for 5 to 7 days, during which the virus is strong, but the patient’s vital energy is not declining, approaching the turning point. Early control can directly lead to the recovery. Therefore, understanding the mechanism of fever is crucial for the diagnosis, treatment, and prognosis of COVID-19 patients.

#### Material Basis of Fever

Fever is usually caused by the interaction of immune cells with exogenous pyrogen and endogenous pyrogenic cytokines. The peripheral fever signal is transmitted to the central temperature regulating center through humoral and neural pathways, thus producing fever (Zeisberger, 1999). Exogenous pyrogen refers to microorganisms and their metabolites from outside, and also the most common fever activators, mainly including bacteria, viruses, fungi, parasites, mycobacteria, *etc.* (Laupland, 2009). The currently recognized major pyrogenic cytokines are interleukin-1 (IL-1), interleukin-6 (IL-6), and tumor necrosis factor-α (TNF-α) (Prajitha et al., 2018).

IL-1 represents a family of two agonists (IL-1α and IL-1β) (Conti et al., 2004). Numerous studies have demonstrated the capacity of peripherally administered IL-1α and IL-1β to evoke fever in a variety of species (Kluger, 1991; Dinarello, 1996). The current explanation for this is that IL-1 induces intermediates, prostaglandin E2 (PGE2), and cyclooxygenase 2 (COX-2), which are considered necessary downstream events which mediate peripheral IL-1-induced fever (Li et al., 2001; Ching et al., 2007). Receptors for IL-6 exist in two forms, a soluble receptor, sIL-6R, and a membrane bound receptor, IL-6R (Vallières and Rivest, 1997). Injection of IL-6 into lateral ventricle can upregulate COX-2 (Cao et al., 2001), increase the level of PGE2 in CSF, and produce fever (Dinarello et al., 1991). Recent research further confirms this view that the pyrogenic effect of IL-6 is exerted by its binding to IL-6 receptors on brain endothelial cells, and that the ligand binding in turn leads to induced expression of the prostaglandin synthesizing enzyme COX-2 *via* intracellular signaling involving the STAT3 pathway (Eskilsson et al., 2014). Intravenous administration of recombinant human TNF (rh TNF) into rabbits can cause fever, and also reveals that the pyrogenic potential of rh TNF is correlated with increased production of PGE2 (Nakamura et al., 1988). TNF-α is the first member of the LPS-induced cytokine cascade to appear following the injection of this exogenous pyrogen (Roth et al., 1998). Again, the mechanism is related to glutathione. It has been shown that the regulation of TNF-α biosynthesis induced by LPS is redox sensitive and requires the participation of the glutathione mediated signaling pathway. In the presence of glutathione, it can activate the activity of PGE synthase-1 (mPGES-1) to produce PGE2 (Wrotek et al., 2015).

#### The Humoral Transmission Pathway of Fever Signal

As mentioned above, the production of PGE2, a common connection has been found in the three kinds of important pyrogenic cytokines. Therefore, PGE2 is considered as the final medium of fever (Roth and Blatteis, 2014). It has been shown that PGE2 from peripheral or central all cause fever (Romanovsky et al., 1999; Blatteis et al., 2000). The pyrogenic cytokines released in the blood by exogenous pyrogen stimulation may play a role outside the brain by binding and activating the cytokine receptor on the capillaries located in the periventricular organs, thus leading to the release of PGE2 (Blatteis, 2006). In addition, in this pathway, the fever signal can also be carried by the PAMPS. The circulating PAMPs represented by LPS release PGE2 through arachidonic acid pathway by binding and activating TLR-4 on the capillaries of the organs around the central chamber of the BBB, and then activate the thermal neurons in the front of the hypothalamus, causing fever (Steiner et al., 2006; Turrin and Rivest, 2004). The synthesis of PGE2 is related to the activation of NF-κB or STAT3 in brain endothelial cells (Nadjar et al., 2005; Rummel et al., 2006).

#### The Neural Transmission Pathway of Fever Signal

**Figure 2:**
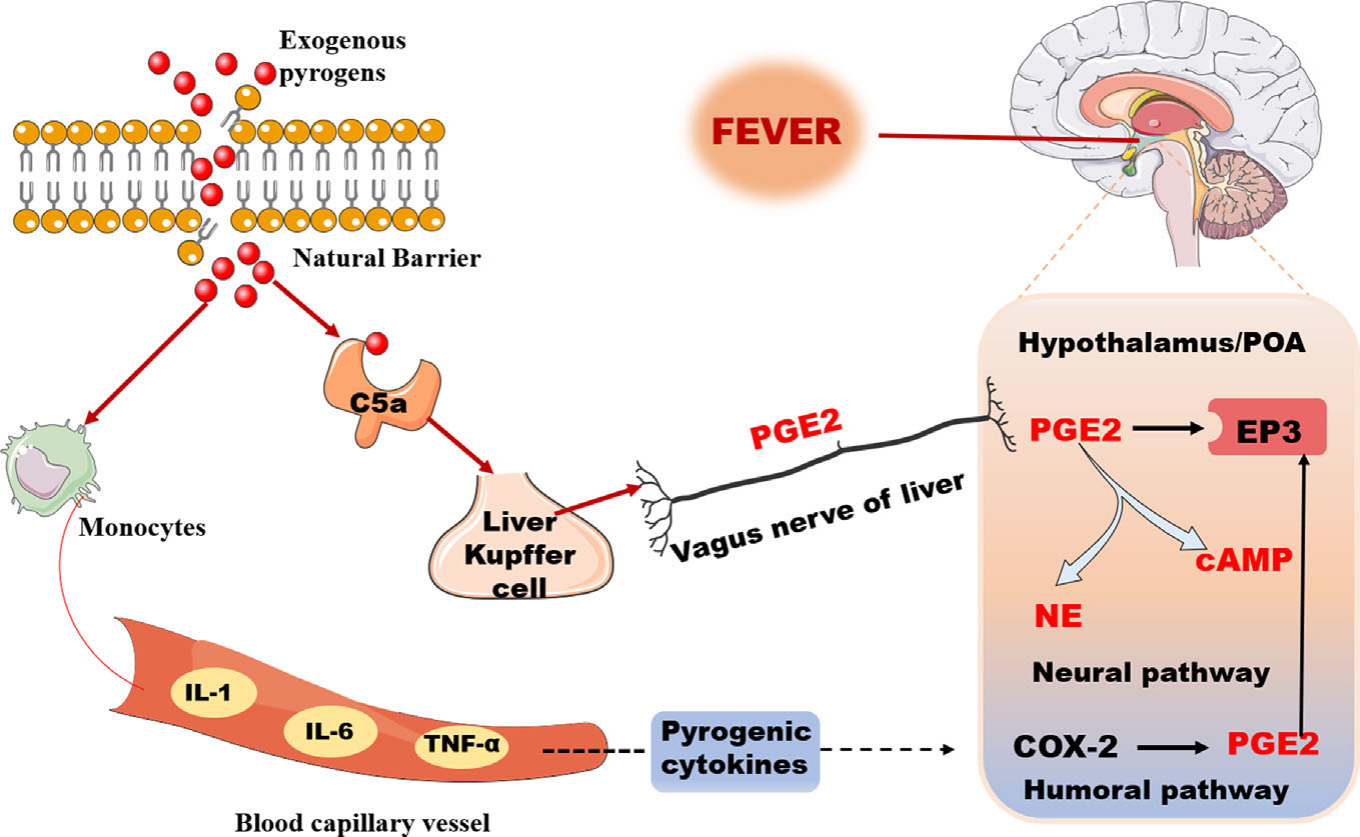
The transmission pathway of fever signal: Humoral pathway and Neural pathway.

The characteristics of febrile reactions are early rapid reaction and late delayed reaction. The activation of the neural pathway is believed to be another mechanism by which fever is rapidly initiated (Roth and De Souza, 2001). It has been shown that the activation of complement component 5A (C5a) immediately triggered the release of PGE2 from Kupffer cells (KC) after LPS injection (Perlik et al., 2005). PGE2 stimulates homologous receptors on the afferent vagus of the liver and binds to EP3 receptors in the OVLT/POA region, resulting in fever (Oka, 2004). PGE2 can also enhance the release of neurotransmitters, especially cAMP released by hypothalamic cells that can change the temperature setting (Dinarello, 2004). Therefore, we believe that PGE2, a rapid fever signal, plays a triggering role in the initial stage of fever through the neural pathway, while the fever signal in the humoral pathway plays a more important role in maintaining fever (**Figure 2**).

### Treatments

Fever is caused by the interaction of immune cells with exogenous pyrogen and endogenous pyrogenic cytokines. The most widely studied pyrogenic cytokines include 1L-1, IL-6, TNF-α, IFNs, and CNTF. After the virus and other pathogens infect the body, they can activate NF-κB to cause the release of TNF-α, IL-1, IFNs, chemokine, etc., which can mediate the aggregation and infiltration of a large number of immune cells into the lung tissue, activate the signal transduction pathway in the cells, start the cascade reaction of waterfall inflammation, release the amount of cytokines, and continuously activate more inflammatory cells to form a vicious cycle. It eventually leads to a cytokine storm. Chen et al. (2020) analyzed 99 confirmed cases of COVID-19, and proposed that the virus spreads through respiratory mucosa to infect other cells, induce a cytokine storm *in vivo*, and produce a series of immune responses. Therefore, it is essential for in the treatment of COVID-19 to inhibit excessive immune cell activation and cytokine production. TCM and its preparations can achieve an antipyretic effect by inhibiting 1L-1, IL-6, TNF-α, and other pyrogenic cytokines, and also can indirectly achieve the effect of initial treatment of COVID-19 by inhibiting the cytokine storm.

#### Single Chinese Herbal Medicine

##### Gypsum Fibrosum

Gypsum Fibrosum is a variety of fibrous crystalline aggregate of hydrous calcium sulfate. Although its main component is hydrous calcium sulfate, it also contains inorganic elements such as sodium, magnesium and iron. Wang et al. (2009) studied the antipyretic activity of Gypsum Fibrosum by intraperitoneal injection of LPS in rats. The body temperature of rats with fever decreased significantly after the intragastric administration of Gypsum Fibrosum extract (0.8 g/ml), indicating that it exerts an antipyretic effect; furthermore, it was speculated that its active components may be the inorganic elements. Calcium is the main ion component of Gypsum Fibrosum. Following the action of gastric acid, part of the gypsum decoction can be transformed into soluble calcium, which can then be absorbed into the bloodstream through the intestines, increasing the calcium ion concentration in the blood, so as to regulate the temperature center and relieve the fever. Zhou et al. (2012) injected a 15% yeast suspension subcutaneously into rat backs, resulting in fever. The rats were infused with gypsum suspension for 7 days (10 g/kg). The results showed that gypsum could play an antipyretic role by reducing the synthesis of PGE2. Gypsum Fibrosum is usually used with Anemarrhena Rhizoma in clinic as antipyretic. One of the most classical prescriptions is Baihu Decoction. When they are used together, it can enhance the dissolution rate of calcium ion and the antipyretic effect of Gypsum Fibrosum (Jia et al., 2013).

##### Bupleuri Radix

Bupleuri Radix is the dry root of *Bupleurum chinense* DC and *Bupleurum scorzonerifolium* Willd. Phytochemical studies reveal that this plant contains essential oils, triterpenoid saponins, polyacetylenes, flavonoids, lignans, fatty acids, and sterols (Yang et al., 2017). In the fight against SARS, it once appeared on the treatment list and attracted scientific attention (Zhao et al., 2007). Bupleuri Radix has good antipyretic effect and has been widely used in clinic. The main components of essential oil and saikosaponin play the role of antipyretic. Chen et al. (2010) injected ET into the normal rabbit to induce fever. The essential oil was extracted from Bupleuri Radix as raw material to prepare the gel, which was sprayed into the nasal cavity of the febrile rabbits. Once 0.2 ml was given, the temperature dropped 0.5°C after 5 h, and the temperature decreased by 0.8°C after 24 h. The results showed that the essential oil of Bupleuri Radix had an antipyretic effect which could play an antipyretic role by reducing the concentration of cAMP in the cerebrospinal fluid of febrile rabbits. Jin et al. (2014) found the antipyretic effect of essential oil and saikosaponin of Bupleuri Radix. The results demonstrated that the antipyretic effect of the treatment group was significant as compared with the control group. Some studies have shown that saikosaponin can significantly reduce the expression of TNF-α, IL-1β, IL-6, and other cytokines. It can also inhibit the NF-κB signaling pathway by inhibiting the phosphorylation of extracellular signal regulated kinase (downstream of TNF-α) (Kim et al., 2015). In conclusion, Bupleuri Radix may play an antipyretic role by reducing cAMP concentration and inhibiting the expression of TNF-α, IL-1β, IL-6, and the NF-κB inflammatory signaling pathway. However, Bupleuri Radix could lead to hepatotoxicity in high doses and with long-term use (Yang et al., 2017).

The commonly used Chinese herbal medicines as antipyretics are listed in **Table 3** in addition to the above.

**Table 3:** The commonly used Chinese herbal medicines as antipyretics.

Chinese herbal medicine	Bioactive components	Model	Treatment	Mechanisms	The species investigated		Result	References	Qualityof evidence
Cinnamomi Ramulus	Essential oil, organic acids, triterpenoid saponins, coumarins, tannins, flavonoid glycosides and polysaccharides	The dorsal root ganglion (DRG) of newborn rats was stimulated at different experimental temperatures	Cultured cells of DRG neurons were incubated with cinnamaldehyde of different concentrations for 12 h	Cinnamaldehyde upregulates the expression and function of Transient receptor potential vanilloid 1 (TRPV1) in DRG neurons through non TRPA1 pathway		New-born SD rats	Cinnamaldehyde, the extract of Cinnamomi Ramulus, has a significant antipyretic effect	(Sui et al., 2010)	IV
Lonicera Japonica Flos	Organic acids, essential oil, flavonoids, triterpenoid saponins	Fever caused by intravenous injection of IL-1β (100 ng)	Intravenous Jin-Yin-Hua injection 1 ml	Inhibition of EP3 expression in preoptic anterior hypothalamic neurons, thus inhibiting the production of PGE2		Healthy New Zealand rabbits	Lonicera Japonica Flos has a significant antipyretic effect	(Xie et al., 2009)	III
Scutellariae Radix	Flavonoids, essential oil, terpenes	Fever caused by intravenous injection of LPS (2 mg/kg)	Baicalin (2 mg/kg, 10 mg/kg, 20 mg/kg) was injected randomly into rabbits	Reduce the excessive production of TNF-α and glutamate; Inhibition of NMDA receptor dependent hydroxyl radicals and PGE2 pathway		Healthy rabbits	Scutellariae Radix has a significant antipyretic effect	(Tsai et al., 2006)	II
Bubali Cornu	Protein, polypeptide and amino acid	Fever caused by subcutaneous injection of 20% yeast (10 ml/kg)	400 mg/kg Bubali Cornu powder extract was administrated orally with a dosage of 10 ml/kg	Change the metabolism of uric acid and cysteine; enhance the activity of antioxidant enzymes; reduce the level of TNF-α; reduce the ROS production and PGE2 synthesis		Aged SD rats (200 ± 20 g)	Bubali Cornu has a significant effect of fever induced by yeast.	(Liu et al., 2016)	II

#### Chinese Patent Medicine

##### Shuang-huang-lian

Shuang-huang-lian series preparations are made from Lonicera Japonica Flos (Jin-yin-hua), Scutellariae Radix (Huang-qin), and Forsythiae Fructus (Lian-qiao). The existing clinical randomized controlled trials demonstrate that Shuang-huang-lian preparations exhibit a certain antipyretic effect. Although it contains a large number of active ingredients, only chlorogenic acid, baicalin, and forsythin have been officially included in the quality control standard (Gao et al., 2014). Baicalin is a type of flavonoid extracted from Scutellariae Radix, which has an obvious antipyretic effect. *In vivo* studies demonstrated that the antipyretic effect of baicalin was related to a decrease in TNF-α, IL-1 β, IL-6, and other cytokines in serum, hypothalamus, and CSF (Li and Ge, 2010).In addition, baicalin inhibited the LPS-modulated upregulation of TLR4 mRNA and protein expression and TNF-α and IL-1β mRNA expression in rats, and downregulated NF-κB activation with simultaneous decreases in TNF-α and IL-1β protein expression (Ye et al., 2015). Forsythoside A (FTA), a monomer of phenethyl alcohol glycosides extracted from Forsythiae Fructus. A previous study suggested that FT-A significantly downregulated TRPV1 expression in the hypothalamus and DRG of yeast-induced pyrexia mice. TRPV1 is a non-selective cation channel gated by noxious heat, playing major roles in thermoregulation. FT-A alleviated fever of yeast-induced pyrexia mice *via* suppression of TRPV1 expression and activation, inhibition of MAPKs, activation of the hypothalamus and DRG, and subsequently decreased secretion of pyretic cytokine as PGE2 and IL-8 (Liu et al., 2017).In addition, FT-A can significantly enhance the phagocytic function of macrophages in LPS-stimulated ra-w264.7 cells and reduce the secretion of TNF-α (Guan et al., 2013). FT-A can also inhibit TNF-α and NF-κB by blocking the LPS/TLR4 signaling pathway (Zeng et al., 2017). It is suggested that FT-A can also inhibit the LPS/TLR4 signaling pathway and reduce TNF-α secretion in order to achieve an antipyretic effect. In a randomized controlled trial to systematically evaluate Shuang-huang-lian injection in the treatment of acute upper respiratory tract infection, it was found that Shuan-ghuang-lian can significantly reduce the fever caused by acute upper respiratory tract infection (Zhang et al., 2013).In addition, Shuang-huang-lian is widely used in the clinical treatment of infectious diseases such as pneumonia, influenza, acute tonsillitis and acute pharyngitis (Song et al., 2000; Chen et al., 2002).

The commonly used Chinese patent medicine as antipyretics are shown in **Table 4**.

**Table 4:** The commonly used Chinese patent medicines as antipyretics.

Chinese patent medicine	Formation	Model	Treatment	The species investigated	Mechanisms	Result	References	Quality of evidence
Qingkailing injection (QKLI)	Gardeniae Fructus, Bubali Cornu, Margaritifera Concha, Isatidis Radix, Lonicera Japonica Flos, Baicalin, Cholic acid	Fever caused by subcutaneous injection of 20% yeast (15 ml/kg)	4.2 ml/kg QKLI into tail vein of rats	Aged SD rats	Decrease the expression of 5-HT and the concentration of 4-aminobutyric acid; improve the metabolism of amino acids and the urea cycle	QKLI has an antipyretic effect	(Gao et al., 2013; Zhang et al., 2017)	II
Jinxin oral liquid (JXOL)	Ephedrae Herba, Descurain Semen, Mori Cortex, Armeniacae Semen Amarum, Gypsum Fibrosum, Peucedani Radix, Scutellariae Radix, Polygoni Cuspidati, Rhizoma et Radix	Fever caused by subcutaneous injection of 20% yeast (15 ml/kg)	Subcutaneous injection of 7.02 g/kg JXOL	Aged SD rats (80 ± 20 g)	Reduce the production of IL-1β, PGE2 and the level of quinolinic acid and pantothenic acid, regulate the metabolism level of 3-phosphoglycerate, pyruvate and other metabolites	JXOL has an antipyretic effect on fever rats	(Qian et al., 2019)	II
Yin Qiao San (YQS)	Lonicera Japonica Flos, Forsythiae Fructus, Platycodonis Radix, Menthae Haplocalycis, Herba, Sojae Semen Praeparatum, Lophatheri Herba, Arctii Fructus, Schizonepetae Herba, Phragmitis Rhizoma, Glycyrrhizae Radix et Rhizoma		weight <20 kg, 1 g/8 h; 20 kg < weight <40 kg,1.5 g/8 h; weight >40 kg,3 g/8 h	21 fever patients		YQS can effectively treat upper respiratory tract infection and fever without serious adverse reactions.	(Liew et al., 2015)	Ic

## Cough

### The Pathophysiological Mechanism of Cough

Cough is a common respiratory disease and one of the early symptoms of bronchitis, pneumonia, asthma, and pertussis (Swarnkar et al., 2013). It is a natural protective mechanism, which helps to clear the secretion of the respiratory tract and prevent harmful particles from entering the respiratory system (Song et al., 2015). From another perspective, cough is one of the ways to enhance the transmission of the virus to the next victim, so inhibiting cough can help reduce the transmission between people (Morice et al., 2015). Cough is usually divided into three types: acute cough (lasting for less than three weeks), subacute cough (lasting for three to eight weeks), and chronic cough (persistent greater than eight weeks) (Kim et al., 2016). Acute cough is commonly associated with viral upper respiratory infection (O’Connell, 1998). Acute infection and inflammation (such as bronchitis or pneumonia) may cause dry cough in some cases (Urso and Michaels, 2016). The cough caused by the COVID-19 lasts for less than 3 weeks, and most of them do not produce sputum, which is actually an acute dry cough.

In general, cough is characterized by changes in the normal respiratory pattern caused by reflex, which is mediated by stimulation of extrapulmonary vagal afferent nerves and the brainstem (Mahashur, 2015). The pathophysiological mechanism of a cough may be related to the following two aspects: sensitizing cough receptors by increasing inflammatory mediators, such as bradykinin, tachykinin or prostaglandin, these sensitive cough receptors will cause an increase in the cough reflex; inducing or enhancing cough sensitivity by contraction of bronchial smooth muscle. The molecular mechanism involved in the former may be regulated by a series of G-protein coupled receptors (GPCRs). The activation of GPCRs has both inhibitory effects (e.g. β2-adrenoceptor and cannabinoid receptors), and excitatory effects (e.g. EP3 and bradykinin B2 receptors) on sensory nerves and the cough reflex (Maher et al., 2011). Prostaglandin E2 and bradykinin can activate airway sensory nerve through EP3 and B2 receptors, and mediate its action through TRPV1 and TRPA1 receptors. The activation of β2-adrenergic and cannabinoid CB2 receptors can inhibit sensory nerves and cough. The main receptors associated with cough reflex are shown in **Table 5**.

**Table 5:** The main receptors associated with cough reflex (Dicpinigaitis, 2004; Maher et al., 2011).

Receptors that excite the cough reflex	
TRPV1	Peripheral pain-sensing neurones and throughout the central nervous system A member of transient receiver potential (TRP). It can respond to various harmful stimuli such as capsaicin, PGE2 and LTB4, which may lead to airway hyperresponsiveness and increase cough sensitivity
Endogenous cannabinoids	
Tachykinin receptor	The tachykinins include substance P, neurokinin A, neurokinin B, and calcitonin gene-related peptides and other neuropeptide transmitters. Neurokinin may induce bronchial hyperresponsiveness, neurogenic inflammation and cough
Bradykinin receptor	
5-HT receptor	Central nervous system
Eosinophil	Eosinophilic airway inflammation is an important cause of chronic non-asthmatic cough
Receptors that inhibit the cough reflex	
Opioid and opioid-like receptor	Central nervous system Antitussive effects mainly mediated by μ- and κ-opioid receptors
Gamma-aminobutyric acid (GABA)-B receptor	
β_2_-adrenoceptor	
Potassium channel	Peripheral nervous system The activation of potassium channels can inhibit the activity of airway sensory nerve, and the regulation of these channels can alleviate cough

Postinfectious cough may occur in the following three ways: 1) The viral infection causes dripping of nasal secretions or produces inflammatory mediators, which lead to an inflammatory reaction of the bronchial mucosa. The inflammatory mediators act on the sensory nerve endings of the airway, increasing the sensitivity of the cough receptors. 2) The virus increases the activity of neuraminidase, destroys the cholinergic M receptor, reduces the affinity with the M receptor, and finally leads to the hyperfunction of cholinergic nerve, which increases the airway responsiveness. 3) By upregulating the expression of neuropeptides, the virus induces neurogenic inflammation, which affects the excitability of afferent nerves and indirectly stimulates cough receptors (McGarvey et al., 2014; Schnoeller et al., 2014). Therefore, the treatment of a cough after infection requires controlling airway inflammation, and reducing airway hyperresponsiveness and cough sensitivity.

### Treatments

Cough is not only one of the main symptoms of COVID-19, but also one of the main routes of transmission of SARS-CoV-2. The virus causes upper respiratory tract infections and pulmonary inflammation, which results in coughing. Many Chinese herbal medicines like Glycyrrhizae Radix et Rhizoma, Asteris Radix et Rhizoma, Farfarae Flos not only have excellent antitussive effect, but also have anti-inflammatory activity. They may reduce airway or pulmonary inflammation by mediating inflammation-related pathways such as NF-κB and reducing airway inflammatory factors. There is a strong association in hyperinflammatory responses in patients with severe COVID-19 infection, so the intervention of these the TCM substance in the early stage of COVID-19 may prevent the disease from mild to severe. TCM possesses the potential effect of synergistic treatment of COVID-19 through multi-components, multi-targets, and multi-ways, which is also in line with the concept of holistic treatment of TCM.

**Figure 3:**
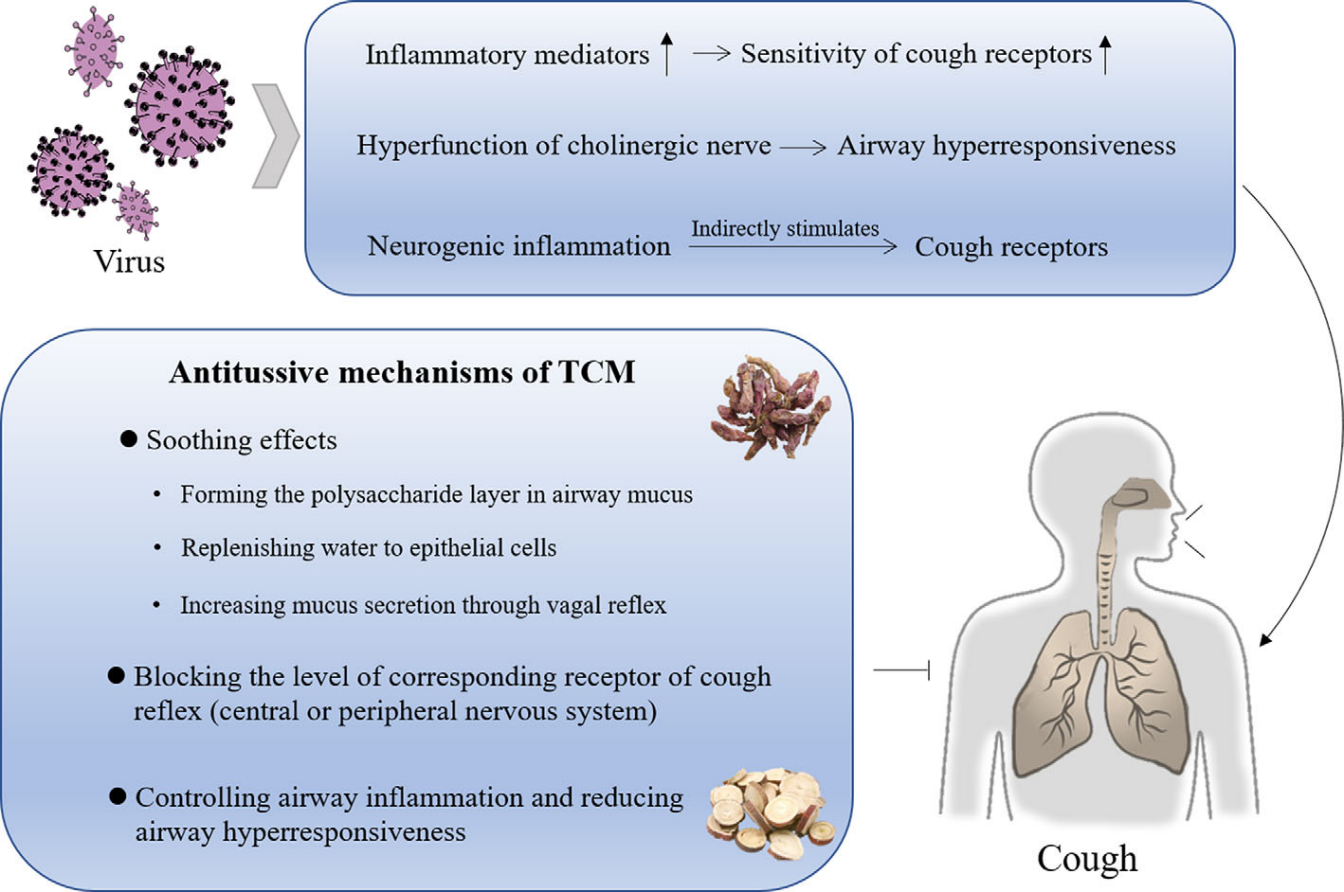
Possible pathogenesis of postinfectious cough and antitussive mechanism of TCM.

The treatment of a cough includes: blocking the level of corresponding receptor of the cough reflex; covering the irritated mucosa in the mouth and throat with mucilaginous herbs, and protecting cells from local stimulation of the mouth or throat, so as to relieve the cough reflex (Fink et al., 2018); controlling airway inflammation and reducing airway hyperresponsiveness (**Figure 3**).

#### Single Chinese Herbal Medicine

##### Glycyrrhizae Radix et Rhizoma

Glycyrrhizae Radix et Rhizoma, the dried root and rhizome of the legume *glycorrhiza urensis* Fisch., *glycorrhiza inflata* Bat., *glycorrhiza glabra* L., is one of the oldest and most popular herbs in the world, exhibiting anti-inflammatory, antiviral, antibacterial, antioxidant, and anticancer activities, immunomodulatory pharmacological activity. It is commonly used in the treatment of cough and lung disease, bronchitis, and gastric ulcer (Pastorino et al., 2018). It contains mainly flavonoids, triterpenoid saponins and glycosides (Hosseinzadeh and Nassiri-Asl, 2015). Among them, the active components with an antitussive effect may be liquiritin apioside, liquiritin, liquiritigenin, 18β-glycyrrhetinic acid and its derivatives, and polysaccharides (Anderson and Smith, 1961; Kamei et al., 2005; Kuang et al., 2018). It has been reported that 50% methanol extract (100 mg/kg) and 70% ethanol extract (800 mg/kg) of Glycyrrhizae Radix et Rhizoma have significant inhibitory effects on the cough reflex in a capsaicin-induced guinea pig cough model and sulfur dioxide gas-induced mouse cough model respectively (Kamei et al., 2003; Jahan and Siddiqui, 2012). In addition, the antitussive effect of the water-extracted polysaccharide fraction (arabinose (52%), galactose (22%), rhamnose (6%) and fucose (2%)) in the citric acid-induced cough model of guinea pigs was even stronger (81%) than that of codeine (62%) at a dose of 50 mg/kg (Saha et al., 2011; Nosalova et al., 2013). Mucus and bioadhesion may be two of the reasons behind its pharmacodynamic effect. The water-extracted polymer fraction of Glycyrrhizae Radix et Rhizoma exhibits bioadhesion to the epithelial mucosa, forming the polysaccharide layer in airway mucus, protecting cells from local oral or pharyngeal stimulation, indirectly affecting the sensitivity of cough receptors and inhibiting cough. Polysaccharides also have the ability to replenish water to epithelial cells, reducing dry cough and supporting phlegm, and increasing mucus secretion through vagal reflex (Nosalova et al., 2013). In addition, it was shown that the cough suppressant effect of liquiritin apioside may depend on the peripheral (regulation of the ATP sensitivity K^+^ channel) and the central mechanism (regulation of the 5-HT system), a possible additional pathway for the cough suppressant effect of Glycyrrhizae Radix et Rhizoma, which may be another way for Glycyrrhizae Radix et Rhizoma to play an antitussive role (Kamei et al., 2003).

##### Asteris Radix et Rhizoma

Asteris Radix et Rhizoma consists of the dried roots and rhizomes of *Aster tataricus* L. f. In the clinical application of TCM, it has been proved to be an effective medicine for the treatment of phlegm cough disease, which has a history of thousands of years. Aster extract exhibits anti-inflammatory, anti-oxidation, anti-tumor and other biological activities. Asteris Radix et Rhizoma is rich in chemical components, including terpenes, flavonoids, sterols, cyclopeptides, *etc.* (Xu et al., 2013). Among them, the main active components of antitussive effect may be asterone, episorbitol, caffeoylquinic acids, astersaponins, aster peptides (Yu et al., 2015), luteolin, and quercetin (Yang et al., 2016). The antitussive effect of Asteris Radix et Rhizoma has been reported many times. Ren et al. observed the antitussive effect of different polar segments of Asteris Radix et Rhizoma through ammonia liquor-induced mice cough model, and the results showed that petroleum ether group, final mother liquor group and 75% ethanol group (5 g/kg) could prolong the latent period of mice cough, inhibit the frequency of cough within 2 min, and n-butanol group had significant antitussive effect, which indicated that aster Asteris Radix et Rhizoma had antitussive effect (Ren et al., 2015). Recently, it was found that in an ammonia-induced mouse cough model, the cough frequency of mice treated with 50% ethanol fraction (40 and 80 mg/kg) eluted from 70% ethanol extract was significantly reduced by 42.9% and 56.5% (both (p <0.001), cough latency increased by 50.5%, 70.9% (both p <0.01). Through further analysis, it is speculated that eliminating or reducing tracheal inflammation (a major source of cough and sputum) through the TLR4-mediated NF-κB pathway may be the mechanism behind its antitussive effect (Yu et al., 2015).

##### Farfarae Flos

Farfarae Flos, flower bud of *Tussilago farfara* L., is widely used in the treatment of cough, tuberculosis and other diseases (Zhao et al., 2014). Farfarae Flos possesses a variety of pharmacological activities, such as anti-inflammatory, antioxidant, anticancer, neuroprotective activities (Lee et al., 2014; Lee et al., 2018). Extensive phytochemical studies have shown that Farfarae Flos contains a large number of components, including volatile oils, phenolic acids, sterols, alkaloids, terpenes, *etc.* Besides, alkaloids, flavonoids, terpenes and saponins are considered to exert antitussive effects (Han et al., 2016). A series of studies have found that the monomeric components with antitussive bioactivity may be 4,5-O-dicaffeoylquinic acid, caffeic acid, chlorogenic acid, 3,5-O-dicaffeoylquinic acid, 3,4-O-dicaffeoylquinic acid, rutin, kampferol analogues, 2,2-dimethyl-6-acetylchromanone, tussilagone, Bauer-7-ene-3β,16α-diol, β-sitosterol, and sitosterone (Li et al., 2012; Wu et al., 2016; Li et al., 2018a; Yang et al., 2020). The antitussive effect of Farfarae Flos has been reported in an animal cough model induced by ammonia. Its aqueous extract at the dose of 2.8 g/kg significantly prolonged the latent period of expectoration and reduced the cough frequency in mice (Li et al., 2012). Further study found that the antitussive effect of Farfarae Flos may be related to four pathways, including the alterations of valine, leucine and isoleucine biosynthesis, pyruvate metabolism, glycerolipid metabolism, phenylalnine, tyrosine and tryptophan biosynthesis, and the imbalance of these pathways is related to a variety of neurological and inflammatory diseases (asthma, emphysema, chronic obstructive pulmonary disease (COPD)) (Li et al., 2018a). In addition, caffeoylquinic acid in Farfarae Flos may inhibit the release of PGE2 in raw 264.7 cells and mediate the cough response by inhibiting leukocytosis or decreasing LPS induced up regulation of COX-2 protein and mRNA levels (Wu et al., 2016). What is more, a network pharmacology study has found that the active components of Farfarae Flos involve 18 targets such as interleukin-2 (IL-2), COX-2, human ribonuclease A3 (RNase3), and biological processes and metabolic pathways related to signal transduction, inflammation and energy metabolism (Li et al., 2018b). These researches have provided a scientific basis for further elaboration of the mechanism of cough and phlegm elimination of Farfarae Flos. Farfarae Flos is safe and effective in the traditional dose range, but the potential toxicity due to the emergence of pyrrolidine alkaloids also needs to be paid attention (Liu et al., 2020).

The commonly used Chinese herbal medicines for cough are listed in **Table 6** in addition to the above.

**Table 6:** Frequently used Chinese herbal medicines for cough.

Chinese herbal medicine	Bioactive components	Model	Treatment	The species investigated	Mechanisms	Result	References	Quality of evidence
Armeniacae Semen Amarum(its therapeutic applications may be limited by reported toxicity and the presence of cyanogenic glycosides)	Amygdalin	Ammonia liquor induced mice cough	Water extract 2, 4 g/kg for 5 days	ICR mice (18–20 g)	Inhibition of proliferation of tracheal smooth muscle cells; stimulation of β2-adrenergic receptor; amygdalin can decompose under the action of β-glucosidase to produce hydrocyanic acid, which has a certain inhibitory effect on respiratory center, making respiratory movement tend to be quiet, thus relieving cough and asthma	Effective in decreasing cough frequency, prolonging cough latency	(Zhang et al., 2010; Xia et al., 2013)	I, IIa
Stemonae Radix	Croomine, neotuberostemonine, stemoninine, tuberostemonine, protostemonine, stemospironine, maistemonine, tuberostemonine H, stemoninoamide, bisdehydrostemoninine	Ammonia liquor induced mice cough	Total alkaloid extract 0.03 g/ml, 0.6 g/kg	Kunming mice of either sex (18–22 g)	Exerting antitussive effect through central and peripheral pathways	Decreasing cough frequency significantly	(Lin et al., 2006; Lin et al., 2008; Yang et al., 2009; Zhou et al., 2009; Xu et al., 2010)	III, III, III, III, III
Citri Grandis Exocarpium	Naringin	Ammonia liquor induced mice cough	Water extract and 70% ethanolic extract (247, 493, and 986 mg/kg)	NIH mice of either sex (18–22 g)	Anti-inflammation effects (blocking the NF-κB pathway); Peripheral antitussive action, but neither through the sensory neuropeptide system nor through the regulation of ATP sensitive K^+^ channels	Obvious antitussive, expectorant and anti-inflammatory effects. And the activity of 70% ethanol extract is much better than that of water extract, even better than that of positive drugs	(Gao et al., 2011; Luo et al., 2012; Jiang et al., 2014)	I, II
Fritillariae Cirrhosae Bulbus	Imperialine, imperialine–N-oxide, isoverticine, and isoverticine–N-oxid, chuanbeinone, verticinone	Ammonia liquor induced cough	80% ethanol extract 1.2, 3.6, 10.8, and 18.0 g/kg for 3 days	Kunming mice of either sex (18–22 g)	Relaxing the bronchi; increasing respiratory secretions; anti-inflammatory effects	Dose dependence significantly increasing cough latency and suppressed cough frequency in mice Low toxicity	(Wang et al., 2011; Wang et al., 2012; Xu et al., 2019)	I, I, I
Schisandrae Chinensis Fructus	Polysaccharides, lignans (schizandrin, schisantherin A, deoxyschizandrin and γ-schisandrin)	Cough models in guinea pigs induced by cigarette smoke (Chronic cough model) and citric acid (Acute cough model),	Polysaccharide extract SCFP-1 (66.5% glucose and 29.4% arabinose) 250, 500, and 1000 mg/kg for 5 days (acute cough model), 14 days (chronic cough model)	Male Hartley guinea pigs (250–350 g)	Reducing the sensitivity of cough receptors; rehydrating epithelial cells and thereby reducing dry cough; increasing mucus secretion through vagus nerve reflex	Remarkable suppressive effects on cough both in chronic cough model and acute cough model	(Zhong et al., 2016)	I
		A guinea pig model of cough hypersensitivity induced by 14 days	Ethanol extract and ethanol-water extract 1 g/kg for 14 days	Male Hartley guinea pigs (250–350 g)	Reducing the infiltration of neutrophils and inflammatory cells, the content of MDA, TNF - α and IL-8 in lung tissue; inhibiting the proliferation of airway epithelium, smooth muscle thickening, inflammatory cell infiltration, TRPV1 and TRPA1 expression	Reducing the frequency of cough and lung inflammation in guinea pigs with cigarette smoke induced cough hypersensitivity	(Zhong et al., 2015)	II

#### Chinese Patent Medicine

Ma-xing-gan-shi-tang (MXGST) is composed of four Chinese herbal medicines, Ephedrae Herba, Armeniacae Semen Amarum, Glycyrrhizae Radix et Rhizoma, and Gypsum Fibrosum. It is a commonly used antitussive prescription in China. It has been made into a variety of prescription preparations, including Maxingzhike tablets, Maxingganshi soft capsules, Maxingganshi concentrated granules, Maxingzhike syrup, Maxingganshi mixture, *etc.* Ephedrine, amygdalin, ephedrine, glycyrrhizic acid, and amygdalin are considered as the main active components (Wang et al., 2016). A series of clinical studies have shown that MXGST is widely used in the treatment of cough, asthma, pneumonia, COPD, and other diseases (Chen et al., 2013; Wang et al., 2014; Lin S. K. et al., 2016; Liao et al., 2017). In an animal experimental study, Lin et al. adopted citric acid-induced cough in guinea pigs cough model to study the pharmacological effect of MXGST water extract in clinical application, and evaluated its subacute toxicity, and found that MXGST water extract (0.4, 1.0 g/kg) has a significant dose-dependent antitussive effect on guinea pigs, which is a safe and effective traditional Chinese medicine prescription (Lin Y. C. et al., 2016). In addition, this study also proved that MXGST water extract has an antipyretic effect on LPS-induced fever rats, which suggests that MXGST may be a promising drug for the treatment of COVID-19. Further studies have found that the antitussive mechanism of MXGST is related to the partial relaxation of bronchial smooth muscle by blocking the acetylcholinergic receptor and histaminergic receptor (Lin Y. C. et al., 2016). Another study showed that MXGST may stimulate the β2-adrenoceptor of bronchial smooth muscle and has an anti-inflammatory effect that inhibits neutrophils from entering the airway (Kao et al., 2001). In terms of composition, the antitussive mechanism of MXGST may be related to the sympathetic α- and β-adrenoceptors activated by ephedrine alkaloids and amygdalin inhibit the central cough center (Miyagoshi et al., 1986).

The commonly used Chinese patent medicines with antitussive effects are shown in **Table 7**.

**Table 7:** Chinese traditional patent medicines with antitussive effect.

Chinese patent medicine	Formation	Bioactive components	Model	Treatment	The species investigated	Mechanisms	Result	References	Quality of evidence
Suhuang Zhike Capsule	Ephedrae Herba, Perillae Folium, Pheretima, Eriobotryae Folium, Perillae Fructus, Cicadaeperiostracum, Peucedani Radix, Arctii Fructus, Schisandrae Chinensis Fructus	Arctiin, ephedrine, schisandrin, pseudoephedrine, schisandrin B, and 1-caffeoylquinic acid	Postinfectious cough	7–14 days	7 randomized controlled trials involving 573 patients	Reducing airway inflammatory factors; alleviating airway hyperresponsiveness and cough sensitivity; relieving airway inflammation	Effective in the treatment of postinfectious cough in adults No serious adverse events	(Ding et al., 2016)	Ia,
Zhi Sou San	Platycodonis Radix, Schizonepetae Herba, Asteris Radix et Rhizoma, Stemonae Radix, Cynanchi Stauntonii Rhizoma et Radix, Glycyrrhizae Radix et Rhizoma, Citri Reticulatae Pericarpium	Total flavonoids	Cough	3–28 days	46 randomized controlled trials with a total of 4007 participants	Relieving pneumonia and airway mucus obstruction; relaxing bronchial smooth muscle;inhibiting the release of eosinophil	Significantly improving the total effective rate and the pulmonary function No serious adverse events	(Xu et al., 2004; Cheng et al., 2017; Zhen et al., 2018)	Ia, III
Eriobotrya japonica-Fritillaria usuriensis dropping pills	Eriobotryae Folium, Fritillariae Ussuriensis Bulbus, Platycodonis Radix, Pinelliae Rhizoma, volatile oil extracts from Mentha haplocalyx Briq.	Ursolic acid, oleanolic acid, peiminine, platycodigenin, polygalacic acid, guanosine	A network pharmacology approach			Acting on the mitogen activated protein kinase (MAPK) pathway, transforming growth factor (TGF)-beta pathway, focal adhesion, tight junctions and the action cytoskeleton		(Tao et al., 2016)	IIa, II

## Fatigue

### Pathological Process and Possible Pathogenesis

Pathological fatigue refers to fatigue caused by a certain disease, and it is also a symptom of the onset of the disease (Matura et al., 2018). It will cause a sub-healthy state of the decline of the function of body, which should be paid great attention (Wang et al., 2018). This kind of fatigue is common in chronic fatigue syndrome (CFS). Some scholars have proposed several hypotheses about the causes and mechanisms of CFS, such as viral infection, immune dysfunction (Yang et al., 2010), and neuroendocrine system disorders (Norheim et al., 2011; Cho et al., 2013). Among them, virus infection is an important factor causing CFS.

A large number of clinical observations have found that the clinical symptoms of CFS are very similar to the symptoms of viral infections, such as fever, sore throat, and muscle swelling when some CFS patients develop symptoms (Collin et al., 2018). At present, there is no firm evidence that CFS is necessarily related to viral infections. The theoretical basis for CFS caused by viral infections is not sufficient, and experts and scholars have not reached a consensus. However, experts agree on this point that virus infection will further cause an imbalance in the immune system of the body, resulting in damage to the central nervous system and muscle structure (Nair and Diamond, 2015).

#### Pathological Fatigue and Immune Function

Brenu et al. (2010) used flow cytometry and found that compared with normal healthy people, granulocyte respiration broke out in patients with CFS, and natural killer (NK) cell expression of CD56 decreased significantly. Nakamura et al. (Nakamura et al., 2010) found that the anti-inflammatory factor interleukin 10 (IL-10) in patients with CFS is higher than that in normal people and the levels of immunoglobulins IgA, IgG, and IgM are disordered. This suggests that immune dysfunction may be one of the mechanisms of CFS. In addition, FIL-1 is a pro-inflammatory cytokine that contains two receptor agonists that induce the expression of other pro-inflammatory factors: IL-1α and IL-1β (Lampa et al., 2012). Cyclooxygenase-2 (Coxoxy-2) inhibitors and inducible nitric oxide synthase (iNOS) inhibitors are targets for the treatment of pain, depression, and fatigue (Von Ah et al., 2008). IL-1 causes expressions of COX-2 and iNOS (Maes, 2009). Therefore, elevated IL-1 levels may be related to fatigue (Miaskowski et al., 2012). At the same time, it was found that in human and animal models affected by CFS, levels of IL-1, IL-6, and TNF-α were also increased (Reyes-Gibby et al., 2013). These pro-inflammatory cytokines can signal the central nervous system and produce behavioral symptoms such as fatigue.

IL-2 is a multifunctional small molecule protein with high activation. It is an immunoregulatory lymphokine produced by activated CD4^+^ T cells and a small amount of CD8^+^ T cells. It can enhance the activity of NK cells and CD ^+^ T cells, thereby inducing IL-1B (Almeida et al., 2002). The production of receptors produces λ-INF, which maintains the growth of T cells *in vitro* and activates a variety of immune cells. The level of IL-2 reflects the functional status of T cells, and its ability to produce is an important indicator of the immune function of the body’s cells (Hilgers and Frank, 1994). Therefore, a decrease in the level of IL-2 may produce a fatigue state.

#### Pathological Fatigue and Neuroendocrine System Disorders

The occurrence of CFS is also closely related to changes in the neuroendocrine system (You et al., 2011). The clinical manifestations of fatigue, depression, bone, and muscle pain in patients with CFS are similar to those in patients with decreased adrenal function (Klimas and Koneru, 2007). The hypothalamus-pituitary-adrenal (HPA) axis contains neurons that synthesize corticotropin releases hormones (CRH) (Tak et al., 2011). CRH regulates adrenocorticotropic hormone (ACTH) through the pituitary. ACTH stimulates the synthesis of corticosteroids such as cortisol or corticosterone through the adrenal cortex. HPA axis disorders often occur in CFS. Inflammatory mediators cause excessive release of corticosterone, which can cause chronic pain, immunosuppression and chronic fatigue. According to Zhao’s research (Zhao, 2010), chronic compound stimulation can lead to a decrease in 5-HT levels in the hippocampus and occipital cortex, disrupt the balance of the hypothalamus-pituitary-adrenal axis, and disrupt the internal environment and cause CFS (Katafuchi, 2006). In addition, cortisol is one of the main effective hormones of the adrenal system acting on peripheral tissues. It was found that cortisol levels in CFS patients were significantly elevated, and the pathogenesis of CFS was related to abnormal HPA negative feedback regulation or excessive activation (Luo et al., 2019). CFS patients have a low level of serum cortisol during steady state, and often experience physical or emotional stress prior to the onset of the disease, which in turn activates the hypothalamic-pituitary-adrenal axis system, leading to increased release of cortisol and adrenocorticotropic hormone. It affects the immune, nervous and other systems, and then produce fatigue symptoms. Therefore, reducing the release of glucocorticoids such as cortisol by adjusting the HPA axis may alleviate the development of pathological fatigue.

#### Pathological Fatigue and Oxidative Stress

The body will produce a large amount of oxygen free radicals during metabolism, which can attack polyunsaturated fatty acids in biofilms, trigger lipid peroxidation reactions, and thus form lipid peroxides, such as malondialdehyde (MDA), resulting in damage to cells and tissues. The level of superoxide dismutase (SOD) activity indirectly reflects the body’s ability to scavenge oxygen free radicals, while the level of MDA indirectly reflects the severity of the body’s cells attacked by free radicals (Hui et al., 2014). MDA is a lipid peroxide formed by free radical attack on polyunsaturated fatty acids in the biofilm to trigger lipid peroxidation. The increase of free radicals will lead to damage to the integrity of the biofilm, increase permeability of the biofilm, release extracellular of enzymes, make electrolyte imbalance, decrease enzyme activity and cell function, resulting in fatigue (Chen and Yan, 2005). At the same time, the body also has antioxidant systems including: SOD, glutathione (GSH), CAT, *etc.* At present, studies have shown that the mental fatigue of CFS is related to the large amount of oxygen free radicals generated in the brain and the antioxidant system is inhibited (Logan and Wong, 2001). Therefore, it is of great significance to seek ways to improve the antioxidant capacity of the body for the treatment of CFS.

### Treatments

It is known that the early pathogenesis of diseases such as COVID-19, SARS, and MERS are immunodeficiency and excessive oxidative stress. These two factors are the common pathological basis for death. For example, peripheral blood flow cytometry was performed on the lung tissue of COVID-19 dead patients. The results showed that CD4^+^ and CD8^+^ T cells were significantly reduced, T cells were overactivated, and CCR4^+^, CCR6^+^, Th17 in CD4^+^ T cells increased, and CD8^+^T cells are rich in cytotoxic particles, which activate the immune system and induce a large number of immune cells to infiltrate into the lung tissue. Other studies have shown that viral infections can directly lead to increased ROS production in alveolar epithelial cells, GSH, SOD, and glutathione peroxidase (GSH-Px) activity is reduced, causing severe oxidative stress in cells, which further aggravating acute lung injury.

These two factors happen to be the same as the causes of fatigue. We hope that the drugs can also treat coronavirus while relieving fatigue symptoms. Therefore, based on the basic pathophysiological mechanism of COVID-19, this section focuses on summarizing the anti-inflammatory and antioxidant intervention strategies of Chinese herbal medicines to specifically block or reverse its pathological development process. This is of great significance for improving the clinical cure rate and reducing the case fatality rate.

#### Single Chinese Herbal Medicine

##### Astragali Radix

Astragali Radix is the dried root of the legume Mongolian *Astragalus membranaceus* (Fisch.) Bge.var.mongholicus (Bge.) Hsiao or Apodium *Astragalus membranaceus* (Fisch.) Bge. Its main ingredients are saponin, flavonoids, polysaccharides, and amino acids (Zhang H. et al., 2014). Huang et al. (2017) administered 40 male pathologically-fatigued BALB/c mice with astragalus polysaccharides (APS) *via* intragastric administration every morning at 8:00 am for 28 days. The required APS was dissolved in 2.0 mL of normal saline. Chronic fatigue can significantly reduce mRNA levels of mitochondrial fusion-related proteins Mfn-1, Mfn-2, and Opa-1 in mice, while mRNA levels of mitochondrial division-related protein Drp-1 significantly increase, indicating that chronic fatigue can make mice mitochondrial fusion-split imbalance in skeletal muscle, eventually causing mitochondrial dysfunction. APS can improve mitochondrial autophagy in skeletal muscle cells by reducing the level of oxidative stress in tissues. In addition, APS can stimulate the origin of mitochondria, maintain the mitochondrial fusion-split balance, improve mitochondrial dysfunction, and ultimately improve cell energy metabolism, thereby increasing the ability of mice to resist chronic fatigue.

##### Ginseng Radix et Rhizoma

Ginseng Radix et Rhizoma consists of the dried roots and rhizomes of the genus *Panax ginseng* C.A.Mey. The main components of ginseng are saponins, polysaccharides, proteins, volatile oils, amino acids, and flavonoids (Lee et al., 2002). Song (2014) Treated 30 male SD rats after successful CFS modeling with ginsenoside aqueous solution 60 mg/kg/d for 6 consecutive weeks. The results showed that compared with the model group, the SOD and GSH activities in the ginsenoside group were significantly increased, and the MDA content was significantly reduced, with a very significant difference (p <0.01). This shows that the increase of free radicals will lead to the damage of the integrity of the biofilm, the increase of the permeability of the biofilm, the release of enzymes inside and outside the cell, leading to abnormal conditions inside and outside the cell, electrolyte imbalance, and decline in cell function, which will cause fatigue. Therefore, ginsenoside Rg1 is thought to reduce the production of the peroxidation product MDA, increase the activity of antioxidant enzymes, improve the antioxidant capacity of nerve cells, reduce the generation of free radicals, and thus increase the ability to resist CFS (Soares et al., 2007).

The commonly used Chinese herbal medicines for fatigue are listed in **Table 8** in addition to the above.

**Table 8:** Chinese herbal medicines for fatigue.

Chinese herbal medicine	Bioactive components	Model	Treatment	The species investigated	Mechanisms	Result	References	Qualityof evidence
Codonopsis Radix	Codonopsis flavonoids	The mice were placed in a glass tank with a water depth of 30 cm, a diameter of 15 cm, and a water temperature of 27–30°C for 30 minutes for 25 consecutive days	Xinjiang wild Codonopsis flavonoid solution (1 mg/kg), continuous gavage for 25 days	160 Kunming male mice (18–22 g)	Improve SOD vitality and reduce the accumulation of free radicals, which helps to eliminate lipid peroxides in the body, thereby delaying fatigue	Compared with the control group, the serum MDA value of the drug group decreased by 55.65%, and the serum SOD activity increased by 186.91%	(Wang and Yuan, 2012)	I
Ziziphus jujuba	Jujubepolysaccharide(JP)	(1) Electric shock method. (2) Restriction method (2 h each time). (3) Cold water swimming 21°C once a day for 30 min each time. Modeling time is 4 weeks	Intragastric administration (400 mg/kg/d) for 28 consecutive days	40 male SD rats (180–220 g)	Related to regulating the body’s immune function status and reducing the content of related inflammatory factors	Compared with the control group, the drug group significantly increased the spleen index, lowered the serum MDA content, improved the T and B lymphocyte transformation ability, and thereby adjusted the body’s immune ability	(Wang et al., 2015)	I
Epimedii Folium	herbaepimediipolysaccharide(HEP)	(1) Swimming for 4 h each time. (2) Noise: Noisy music is played every day from 8 pm to 8 am the next day. (3) Treadmill exercise for 1 hour (20 m/min) every day. (4) Crowding: Each group of 10 rat lives in a standard feeding cage. The time is 28 days	Inject HEP 100 mg/kg daily for 14 days	50 female SD rats (180–220 g)	HEP indirectly regulates HPA axis function in CFS patients by increasing norepinephrine levels	The weight of rats, the number of crossing the adjacent lattice, and the number of standing (open field test) in the drug group all increased significantly (P <0.01). Both the time to find the platform (Morris water maze) and the time to rest (suspended tail experiment) decreased significantly (P <0.01)	(Chi et al., 2017)	I
Rhodiolae Crenulatae Radix et Rhizoma	SHR-5	(1) Cold water swimming (16 ± 1°C) for 7 min each time (2) Restraint: After the restraint is placed in the rat’s head to the vent for 30 minutes The modeling time is 21 days	Intragastric administration 168 mg/kg daily for 21 consecutive days	40 male SD rats (180–220 g)	IL-2 and TNF-α levels in serum were significantly increased	The differences in the levels of IL-2 and TNF-α in the serum of the model group and the normal group were statistically significant (P <0.05)	(Wang, 2014)	II

#### Chinese Patent Medicine

Angelica Decoction for Replenishing Blood, is composed of Astragali Radix and Angelicae Sinensis Radix in a 5:1 ratio. The primary chemical components of Astragalus are saponins, flavonoids, and polysaccharides; the main chemical components of angelica are volatile oils, organic acids, and polysaccharides. Liu et al. modeled 56 SD male rats (180–220 g) by tying an iron block weighing approximately 10% of the rat’s own weight to its tail, and then placing it into a transparent water tank with a depth of 30 cm, at a constant temperature of 25 degrees Celsius. The rats were forced to swim exhaustively. When the rats’ swimming movements were uncoordinated or their heads sank into the water surface within 10 s, they could not return to the water surface, and the 15.00 g/kg drug was administered to the stomach for 29 consecutive days after modeling successfully. The results showed that compared with the blank group, the Angelica Decoction for Replenishing Blood could significantly reduce the levels of TNF-α and IL-6 in the serum (p < 0.01). This demonstrates that CFS can produce inflammation in the body, and Angelica Decoction for Replenishing Blood can effectively alleviate this situation. At the same time, the activity of SOD in the serum of angelica buxue decoction group increased significantly (p <0.01), suggesting that a large number of free radicals in CFS rats may lead to oxidative stress. Angelica buxue decoction can effectively remove free radicals in the body and alleviate the oxidative stress reaction of the body. Threonine is an essential amino acid. When it is lacking, the synthesis of immunoglobulins and the production of T-lymphocytes and B-lymphocytes will be affected, thereby upsetting the body’s immune functioning. Serine is involved in the production of immune hemoglobin and antibodies, and plays an important role in the maintenance of the immune system. Metabolomics results show that Angelica Decoction for Replenishing Blood can improve the thymic degenerative changes by increasing the levels of threonine and serine, promote the differentiation and maturation of white blood cells, and block the NF-кB, JNK, and p38MAPK signaling pathways to regulate the immune system and improve chronic fatigue syndrome (Liu et al., 2011).

The commonly used Chinese patent medicines for fatigue are shown in **Table 9**.

**Table 9:** Chinese patent medicines for fatigue.

Chinese patent medicine	Formation	Bioactive components	Model	Treatment	The species investigated	Mechanisms	Result	References	Qualityof evidence
Jiawei Sini Powder granules	Radix Bupleuri 10 g, Aurantii Fructus Immaturus 10 g, Radix Paeoniae Rubra 10 g, Glycyrrhizae Radix et Rhizoma Praeparata cumMelle 10 g, Cinnamomi Ramulus 10 g, Acori Tatarinowii Rhizoma 6 g	—	Received various stresses within 49 days, including electroacupuncture (sparse wave, 10 s each time, 5 times each), exhausted swimming, dark box roller (60 r·min^−1^, 10 min), tail suspension (10 min, and gradually extended), sleep deprivation for 24 h, an average of 6 times per stimulation	Dosing was started on the 49 th day after successful modeling, continuous administration for 7 days, each dose was 8.64 g/kg	70 Kunming mice (17–20 g)	Related to regulating the body’s immune function and reducing IL-2 content	Compared with the model group, the quality and behavior changes of the drug group model were statistically significant (P <0.05)	(Zhang T. et al., 2014)	II
Addition and subtraction of Guipi Decoction	Astragali Radix 30 g, Ziziphi spinosae semen 25 g, Codonopsis Radix 15 g, Longan Arillus 15 g, Atractylodis Macrocephalae Rhizoma 15 g, Polygalae Radix 15 g, Angelicae Sinensis Radix 15 g, Glycyrrhizae Radix et Rhizoma 10 g, Aucklandiae Radix 7 g	Astragalus saponin I.V.	—	1 dose daily, 300 ml each morning and evening, 30 days as a course of treatment, a total of 3 courses of treatment	80 patients with CFS	Astragalus saponin IV is the main active ingredient that exerts a positive inotropic effect, which can effectively improve cardiac contraction and diastolic function, and enhance myocardial contractility by inhibiting Na ^+^ - K ^+^ - ATP	The total effective rate in the treatment group was 85.0% (34/40); the control group was only 67.5% (27/40) There was a significant difference between the two groups (P <0.05)	(Ouyang et al., 2018)	Ib
Yishen Buxue Ointment	Angelicae Sinensis Radix 10 g, Rehmanniae Radix 15 g, Radix Paeoniae Alba 10 g, Chuanxiong Rhizoma 10 g, Cuscutae Semen 15 g, Epimedii Folium 12 g, Psoraleae Fructus 10 g, Lycii Fructus 10 g	Tetramethylpyrazine	—	Decoction 300–400 ml, take 2 times in the morning and evening, continuous treatment for 6 weeks	104 patients with CFS	Enhance the immune function of patients, IgG, IgM, IgA levels are significantly increased, and ligustrazine has the effects of scavenging oxygen free radicals, improving blood rheology, regulating lipid metabolism	The total effective rate of treatment in the drug group was significantly higher than that in the control group (P <0.05)	(Yuan, 2018)	Ib

## Diarrhea

### The Mechanism of Diarrhea

Diarrhea, a common digestive disease, is caused by a variety of pathogens and other factors. Diarrhea results from the abnormal absorption or transport of water and electrolytes in the intestine. Any substance, whether infectious biological factor, noninfectious humoral factor, or some drugs, which blocks the active absorption or activation of active secretion of the intestine, will cause diarrhea. Diarrhea is also caused by the increase of osmotic gradient or hydrostatic pressure in intestinal tissue. Infection with bacteria, viruses, or parasites is the main cause of diarrhea, which is also known as infectious diarrhea or gastroenteritis (Thapar and Sanderson, 2004; Schlossberg, 2015). The occurrence and spread of infectious diarrhea are considered to be the results of poor sanitation. Other causes of diarrhea include hyperthyroidism, lactose intolerance, inflammatory bowel disease, drug effects, and irritable bowel syndrome.

Clinically, quite a few patients infected with COVID-19 experienced diarrhea in the early stage or in the course of the disease, which is mostly self-limiting and varies in severity. In the course of treatment, it was also found that diarrhea caused by COVID-19 mainly occurred after antiviral treatment. Infectious virus particles were isolated from feces of some patients, which increased the possibility of feces-oral transmission. The main causes of diarrhea were considered to be gastrointestinal mucosal injury or gastrointestinal dysfunction caused by COVID-19 and adverse reactions caused by the use of antiviral drugs. The mechanism may be related to the rapid and massive production of various cytokines such as TNF-, IL-6, IL-1, and IL-8 in body fluids when patients were infected with COVID-19.

### Treatments

TCM has thousands of years of valuable experience in the treatment of diarrhea. Many antidiarrheal TCM substances, such as Pogostemon Herba, Atractylodis Rhizoma, and Citri Reticulatae Pericarpium, may prevent and treat diarrhea by TNF, IL-6, VIP, and NF-κB. Some of them can also enhance the anti-virus ability of the body by enhancing the immunity, and protect various organs at the same time. Patients with mild conditions can recover quickly by strengthening their own immunity and eliminating the virus by the immune mechanism.

#### Single Chinese Herbal Medicine

##### Pogostemon Herba

Pogostemon Herba is a dry aboveground part of the *Pogostemon cablin* (Blanco) Benth. The chemical constituents of Pogostemon Herba can be divided into two categories: volatile components (patchouli oil) and non-volatile components, including monoterpenes, and sesquiterpenes, flavonoids, organic acids, and alkaloids. A large number of studies showed that the main components of patchouli oil include patchouli alcohol and patchouli ketone. Chen et al. (1998) found that the water extraction and oil-free extraction can inhibit the gastrointestinal propulsion of the normal mice and treat diarrhea in mice caused by Sennae Folium, suggesting that two extractions can inhibit diarrhea by inhibiting the excessive peristalsis of the small intestine. Therefore, the effective component of Pogostemon Herba to improve intestinal function may be water-soluble. Wu et al. (2020) established a rat model of intestinal mucositis *via* intraperitoneal injection of 5-fluorouracil, and intragastrically administrated Patchouli alcohol (PA) (10, 20, and 40 mg/kg) to evaluate the effect of PA on intestinal mucositis. The results showed that PA could effectively improve diarrhea in intestinal mucositis rats, preliminary confirming PA efficacy. Further experiments revealed that PA not only decreased the levels of TNF-α, IL-1β, IL-6, and MPO but also increased the level of IL-10 significantly. In addition, the expression of mucosal barrier proteins and the microbiota community were also improved after PA treatment in diseased rats. Hence, PA may prevent the development and progression of intestinal mucositis by improving inflammation, protecting the mucosal barrier, and regulating intestinal microbiota.

##### Citri Reticulatae Pericarpium

Citri Reticulatae Pericarpium, commonly referred to as “Chen-pi” in Chinese, is an orange-colored *Citrus reticulata* Blanco fruit peel. Up to now, approximately 140 chemical components have been isolated and identified from Citri Reticulatae Pericarpium, including alkaloids, flavonoids, and essential oils. And among them, flavonoids were considered to be the primary bioactive constituents of herbal medicine, mainly including hesperidin and nobiletin. The Citri Reticulatae Pericarpium decoction can alleviate diarrhea of rats caused by Sennae Folium. Guan (Guan et al., 2002) found that the Citri Reticulatae Pericarpium decoction (6.25%, 12.5%, 25%, 50%, 75%, 100%) can significantly inhibit the spontaneous activity of the isolated duodenum of rabbits, reduce the contractility and tension, and show a dose-response relationship. It has an antagonistic effect on the enhancement of ileal contraction induced by acetylcholine, BaCl_2,_ and 5-HT. Moreover, it may further relax the isolated rabbit intestines which first used atropine, epinephrine and dopamine, but the tension decreased. It is suggested that the inhibitory effect of hesperidin on intestinal motility may not be the main component of Citri Reticulatae Pericarpium. The inhibitory effect of Citri Reticulatae Pericarpium is mediated by the cholinergic receptor, 5-HT receptor, or directly on smooth muscle.

##### Atractylodis Rhizoma

Atractylodis Rhizoma is derived from the dried roots of *Atractylodes lancea* (Thunb.) DC. and *Atractylodes chinensis* (DC.) Koidz. The main components in its essential oil are atractylol (a mixture of β-cineole and atractylol), atractylone, atractylon, *etc.* Ancient Chinese doctors thought that Atractylodis Rhizoma could be used for dampness blocking, abdominal distention, diarrhea and so on. Modern research has found that Atractylodis Rhizoma has anti-diarrhea and anti-inflammatory effects. Wang et al. (2002) found that β-cineole can significantly improve the physical signs and inhibit the gastrointestinal movement of spleen deficient mice. β-eucalyptol has an obvious antagonistic effect on the acceleration of gastrointestinal motility induced by neostigmine loaded mice, and also on the gastrointestinal motility induced by Rhei Radix et Rhizoma. Research has shown that (Chen et al., 2018) the N-butanol portion of Atractylodis Rhizoma can significantly improve the level of serum anti-inflammatory factor IL-10 and AQP3 of colon mucosa, reduce the level of TNF-α and diarrhea index, relieve the inflammation of the digestive tract, promote the absorption of water by the colon, and play a role in strengthening the intestine and stopping diarrhea. The results showed that the antidiarrheal effect of Atractylodis Rhizoma was enhanced after frying coke and the N-butanol extract was one of the effective parts of Atractylodis Rhizoma. Shi (Shi et al., 2020) found that the ethanol extract of deep-fried Atractylodis Rhizoma can significantly reduce the level of intestinal inflammatory cytokines, increase the expression of AQP3 and AQP8, and restore the abnormal water metabolism. In addition, it can regulate intestinal flora and improve intestinal structure. The commonly used Chinese herbal antidiarrheal medicines are listed in **Table 10** in addition to the above.

**Table 10:** The single Chinese herbal medicines of antidiarrheal.

Chinese herbal medicine	Bioactive components	Model	treatment	The species investigated	Mechanisms	Result	References	Quality of evidence
Coicis Semen	Fatty acids and esters, coixol, coixan, flavonoids, glycoproteins, sterols, lactams	Rhei Radix et Rhizoma : Magnoliae officinalis Cortex : Aurantii Fructus Immaturus (4:5:3) 1.5 mL/100 g, administered once every other day, fasted on the same day, fed enough and swam to endurance limit on the next day, lasting for 15 days	Coicis Semen decoction high and low dose groups were given 200 g·kg^−1^·d^-1^ and 10 g·kg^−1^·d^−1^ by gavage, respectively, for 10 consecutive days	50 SD rats (200 ± 10 g)	Increasing the levels of serum SP, MTL, GAS, CCK, and SS, reducing the content of serum PP, and then regulating gastrointestinal motility	Rat serum hormones have been improved to varying degrees, the number of stools has decreased, and the stools have changed	(Li and Liu, 2019)	II
Aurantii Fructus Immaturus	Flavonoids, essential oil, alkaloids	Ig Rhei Radix et Rhizoma decoction once a day (8.9 g/kg, calculated by crude drugs), 10 ml/kg, for 14 consecutive days	Ig Aurantii Fructus Immaturus solution 10 mL/kg, once a day for 7 consecutive days	170 SD rats (180–200 g)	Promoting the secretion of serum gastrin, acetylcholine, motilin and inhibiting the secretion of vasoactive intestinal peptide	Promote Gastrointestinal motility of spleen deficiency model rats	(Hu et al., 2017)	II
Arecaesemen	Alkaloids, flavonoids, tannins, fatty acids, terpenes, steroids	Gastrointestinal *in vitro* experiment	The Arecaesemen decoction with 12.5% concentration was added 0.025 ml, 0.05, 0.1, and 0.2 ml in sequence, and the interval was 5 minutes	Isolated stomach from rats	Obviously promoting the contraction of the fundus muscle strips in rats, which is manifested by a marked increase in the baseline tension and a significantly increased amplitude	Promote gastrointestinal movement	(Ni et al., 2003)	IV
Magnoliae Officinalis Cortex	Magnolol, honokiol	Each one was given 0.4 ml castor oil by gavage	Water extracts (100, 200, 400 mg/kg) were perfused into stomach respectively	30 sterile Kunming mice	Magnolol and honokiol may exert anti-diarrheal effects by regulating gastrointestinal motility and inhibiting inflammation in the form of Ca^2+^ antagonists	Significantly reducing the diarrhea rate and diarrhea index of mice, and also inhibiting the frequency of loose stools in mice	(Xie et al., 2017)	II
Amomi Fructus	Essential oil, polysaccharides, flavonoids, organic acids, phenols, inorganic compounds	Intragastric administration of 8% Sennae Folium powder suspension once (0.25 ml/10 g)	The essential oil (2, 1, 0.5 ml/kg) were given by gavage once a day, lasting for 3 days	SPF Kunming mice (20 ± 2 g)	Regulating gastrin and prostaglandin E2(PGE2) secretion and VIP expression	Inhibiting diarrhea in mice caused by Sennae Folium	(Zhao et al., 2009)	I

#### Chinese Traditional Patent Medicine

Huoxiang Zhengqi Oral Liquid is composed of Atractylodis Rhizoma, Citri Reticulatae Pericarpium, Magnoliae officinalis Cortex, Angelicae dahuricae Radix, Poria, Arecae Pericarpium, Pinelliae Rhizoma, licorice extract, patchouli oil, and perilla leaf oil. The active ingredients of Huoxiang Zhengqi Oral Liquid mainly include liquiritin, narirutin, hesperidin, ammonium glycyrrhetate, honokiol, magnolol, thymol, guanosine, adenosine, imperatorin, isoimperatorin. Different Huoxiang-Zhengqi preparations have certain anti-diarrhea effects. Taking Huoxiang Zhengqi Oral Liquid as an example, long-term clinical experience shows that it has a significant effect on improving gastrointestinal symptoms. It was found that Huoxiang Zhengqi Oral Liquid could significantly improve the symptoms of diarrhea in spleen deficient rats with dampness syndrome (Xue et al., 2011). Its mechanism might be related to the increased of the expression of ZO-1 in the ileal mucosa, the regulation of CD4 and CD8 T cells in Peyer’s patch, and the inhibition of TNF-α level in intestinal homogenate (He et al., 2007).

The commonly used Chinese traditional patent medicines of antidiarrheal are shown in **Table 11**.

**Table 11:** Chinese traditional patent medicines of antidiarrheal.

Chinese traditional patent medicine	Components	Model	Drug delivery cycle	The species investigated	Mechanisms	Result	References	Quality of evidence
Wu Ling San	Poria, Alismatis Rhizoma, Polyporus, Cinnamomi Cortex, Atractylodis Macrocephalae Rhizoma	Daily morning gavage: 20 ml/kg Senna solutionfor 6 day	Wu Ling San (1.35, 2.7, 5.4 g/kg) was administered once a day for 7 days	216 SPF grade SD rats, (160 ± 20 g)	Up regulated expression of AQP4 and AQP4mRNA in colon mucosa of diarrhea rat	Compared with the control group, the stool in the model group was still soft	(Liu et al., 2012)	I
Sishen Wan	Myristicae Semen, Psoraleae Fructus, Schisandrae Chinensis Fructus, Euodiae Fructus, Jujubae Fructus	0.2 g/kg of adenine was administered to each stomach for 4 weeks, then 10 mL/kg of ice Sennae Folium water from the third week for 2 weeks	Intragastric administration at 4.2, 3.23, 0.97 g/kg, respectively, once a day for 2 weeks	45 SPF grade SD rats, (140–160 g)	Sishen Wan has the potential as a therapeutic regiment for treatment of diarrhea-predominant irritable bowel syndrome (IBS-D) due to partial regulation of the intestinal flora	Compared with the model group, the diarrhea index decreased and the intestinal sensitivity decreased	(Liu et al., 2019)	III

## Conclusion and Perspectives

The anti-epidemic experience of China shows that it is of great value in the clinical treatment for COVID-19 to intervene early, improve clinical symptoms of patients, block the transition of mild cases to severe cases, shorten the course of the disease and promoting self-recovery, which can minimize the incidence and mortality of severe illness, and make full use of tight and limited medical resources. At present, the focus is on the research of antiviral drugs and vaccines, but there are few reports on treating early mild symptoms. Fever, cough, and fatigue are the most common symptoms of COVID-19, while some special patients experience diarrhea rather than fever in the early stage. Therefore, this paper summarizes the physiological and pathological processes of fever, cough, fatigue, and diarrhea, and explores the material basis, action mechanism and clinical research of Chinese herbal medicines and Chinese patent medicines with corresponding therapeutic effects, in order to provide reference for the efficient use of existing drugs. TCM has the unique properties of multi-components and multi-targets. The majority of these mentioned drugs may not only exert the effects of antipyretic, antitussive, anti-fatigue, and antidiarrheal, but also have the properties of anti-inflammation, antioxidation and immunity enhancement; and some of them are antiviral.

Such rich pharmacological activities are of great benefit in the initial treatment of COVID-19. On the one hand, these medicines may have the ability to relieve symptoms, reduce the rate of infection and prevent the transition from mild to severe in the early stages of infected patients. On the other hand, it is possible to cut down the dosage or use of hormones, decrease the dosage of first-line antiviral drugs or shorten their usage time, so as to minimize the potential damage to the liver by these drugs, and reduce the mortality of critically ill patients through the treatment of integrated traditional Chinese medicine and western medicine. However, these medicines also have shortcomings, mainly manifested in the lack of in-depth research on the material basis and mechanism of action, as well as the imperfection of clinical trials. Therefore, it is urgent to design more stringent controlled clinical trials in order to provide more scientific and reliable evidence for fighting COVID-19 all over the world.

## Author Contributions

D-KZ and LH put forward the idea. C-HL, L-LM, H-ML, and WL gather the materials, and wrote the paper. R-CX, Z-MC, and J-ZL contributed to the revisions. All authors contributed to the article and approved the submitted version.

## Funding

We are grateful to the support of National Nature Science Fund (81773918) and Innovative Research Team Project of Chinese Medicine Discipline in Chengdu University of Traditional Chinese Medicine (CXTD2018006).

## Conflict of Interest

The authors declare that the research was conducted in the absence of any commercial or financial relationships that could be construed as a potential conflict of interest.
